# Systemically delivered *Bacteroides thetaiotaomicron*-derived bacterial extracellular vesicles inhibit primary and metastatic melanoma growth

**DOI:** 10.1128/spectrum.02512-26

**Published:** 2026-09-29

**Authors:** Christopher A. Price, Emily J. Jones, Nilda Ilker, Alicia D. Nicklin, Rokas Juodeikis, Alastair M. McKee, Luke J. Mitchell, Régis Stentz, Lindsay J. Hall, Simon R. Carding, Stephen D. Robinson

**Affiliations:** 1 Food, Microbiome and Health Programme, Quadram Institute Bioscience, Norwich Research Park 7308, Norwich, United Kingdom; 2 Division of Blood and Marrow Transplantation and Cellular Therapy, Department of Medicine, Stanford University School of Medicine 10624, Stanford, California, USA; 3 Microbes, Infection and Microbiomes, College of Medicine and Health, University of Birmingham 1724, Birmingham, United Kingdom; 4 Norwich Medical School, University of East Anglia, Norwich Research Park 6106, Norwich, United Kingdom; 5 School of Biological Sciences, University of East Anglia, Norwich Research Park 6106, Norwich, United Kingdom; Cleveland Clinic Lerner Research Institute, Cleveland, Ohio, USA

**Keywords:** extracellular vesicles, bacteroides thetaiotaomicron, host-microbe interactions, melanoma, microbiome-derived products

## Abstract

The gut microbiome can contribute to anti-tumor immunity and cancer therapy responses, but translating live microbe-based interventions remains challenging due to safety, controllability, and delivery constraints. Bacterial extracellular vesicles (BEVs) are an attractive cell-free alternative, as they package bacterial cargo into a nanoscale format capable of host-cell engagement, immunological activation, and systemic distribution. Here, we investigated the anti-tumor potential of BEVs derived from the human gut commensal *Bacteroides thetaiotaomicron* (Bt). We show that the delivery route is a major determinant of efficacy. Intravenous, but not intraperitoneal, administration produced robust anti-tumor activity in a B16F10 melanoma mouse model. Intravenously delivered Bt BEVs suppressed primary tumor growth in a dose-dependent manner and reduced metastatic outgrowth in the lung. Bt BEVs did not reduce B16F10 metabolic viability during a 24-h *in vitro* exposure, but they activated NF-κB and Toll-like receptor signaling in human reporter systems, and NanoLuc-associated signal from labeled BEV preparations was detected in excised tumor tissue following systemic administration. Although the host pathways responsible for tumor control remain to be defined, these findings identify naturally produced commensal-derived Bt BEVs as a candidate cell-free microbial therapeutic modality for cancer therapy.

**IMPORTANCE:**

Gut bacteria can influence cancer immunity, but using live microorganisms as treatments creates practical and safety challenges. Bacterial extracellular vesicles offer a cell-free way to deliver microbial molecules to the host, yet the anti-cancer potential of vesicles naturally produced by common gut bacteria remains poorly defined. Here, we show that vesicles from the human gut bacterium *Bacteroides thetaiotaomicron* suppress primary melanoma growth and reduce lung tumor burden in mice when delivered intravenously, whereas delivery into the abdominal cavity is much less effective. The vesicle preparations did not reduce melanoma-cell viability directly in culture, suggesting that the *in vivo* effect is not explained by simple toxicity to tumor cells. These findings establish that naturally produced vesicles from a common gut bacterium can exert anti-tumor activity after systemic delivery and identify the administration route as an important determinant of their activity. These findings support their further evaluation as cell-free therapeutic platforms for cancer.

## INTRODUCTION

The intestinal microbiome is emerging as an important modifier of cancer progression and treatment response, with evidence that microbial communities and their products can shape anti-tumor immunity, alter treatment efficacy, and influence toxicity (1–3). Emphasis is now shifting from descriptive taxonomic associations to the identification of defined microbial effectors with demonstrable therapeutic properties (4, 5).

One candidate class of such effectors is bacterial extracellular vesicles (BEVs), which are naturally produced by both Gram-negative and Gram-positive bacteria (6, 7). Bacterial extracellular vesicles carry a range of bacterial cargo, including membrane-associated and cytosolic components, and can interact with epithelial and immune cells at mucosal surfaces in the gastrointestinal tract (GIT) (8–10). This provides a plausible cell-free mechanism by which bacterial products can influence host responses without requiring direct bacterial colonization or invasion. Via the GIT, BEVs can also gain access to the circulatory system to reach distant tissues and organ systems, including the brain (11). In oncology, BEVs have increasingly been explored as immunostimulatory agents, tumor-targeting carriers, and vaccine or drug-delivery platforms (6, 8, 12). However, much of this literature has focused on engineered bacteria and vesicles, or vesicles derived from pathogens, rather than native vesicles released by common commensals. As a result, the anti-tumor potential of naturally produced vesicles from non-pathogenic members of the human microbiome remains less well defined.

*Bacteroides thetaiotaomicron* (Bt) is a prominent human gut commensal Gram-negative bacterium with well-established effects on host physiology and mucosal immune homeostasis (13–15). Bt BEVs are naturally produced in the GIT, can cross the intact intestinal epithelial barrier (11), and can modulate immune (16), epithelial (17), and neuronal cell (18) responses. This supports the idea that biologically relevant host effects can be mediated by secreted non-viable microvesicles rather than by live bacterial colonization alone (16, 17). Thus, Bt is a credible source organism for testing whether native commensal bacteria-derived BEVs can function as non-viable anti-cancer therapeutics.

Despite their promise, several key questions remain unresolved regarding BEVs. It is unclear whether native Bt BEVs can generate anti-tumor effects *in vivo*, whether the route of administration influences efficacy, whether they directly alter tumor-cell viability, and which host pathways mediate any *in vivo* activity. These questions are relevant for clinical translation, as formulation and delivery parameters will be major determinants of success for vesicle-based microbial therapeutics (19, 20).

Here, we evaluated the anti-tumor activity of Bt-derived BEVs in preclinical tumor models, focusing on delivery route and *in vivo* efficacy. We show that intravenous (IV), but not intraperitoneal (IP), administration produces dose-dependent suppression of primary tumor growth and reduced pulmonary lesion outgrowth. We further show that Bt BEVs activate human innate immune reporter systems *in vitro* and that NanoLuc-associated signal from labeled BEV preparations was detected in excised tumor tissue after systemic delivery. Together, these findings position native commensal Bt BEVs as a potential microbial therapeutic modality, identifying delivery route as a critical determinant of biological activity.

## MATERIALS AND METHODS

### Animal studies

All animal procedures were performed in accordance with UK Home Office regulations and the European Legal Framework for the Protection of Animals used for Scientific Purposes (European Directive 86/609/EEC). C57BL/6 mice were maintained at the Disease Modeling Unit, University of East Anglia, under project licence PP8873233. Mice were used at 8–12 weeks of age and were randomly mixed between cages before the start of experiments. Subcutaneous tumor experiments were performed in age-matched male C57BL/6 mice, whereas the B16F10 experimental metastasis model used age-matched male and female C57BL/6 mice.

### Bacterial culture, BEV generation, and quantification

*Bacteroides thetaiotaomicron* VPI-5482 was grown anaerobically at 37°C in Bacteroides Defined Medium (BDM) adapted from Juodeikis et al. (21). This chemically defined medium has previously shown no detectable particulate background by nanoparticle tracking analysis, in contrast to complex BHI medium (19). BEVs were isolated from culture supernatants as previously described by Stentz et al. (22) with minor modifications. Briefly, bacterial cultures were centrifuged at 5,500 × *g* for 45 min at 4°C, and the supernatants were passed through 0.22 μm polyethersulfone membranes (Sartorius). Filtered supernatants were concentrated by ultrafiltration using a 100 kDa molecular weight cut-off device (Vivaflow 50R, Sartorius), washed with 500 mL PBS, and concentrated to 1 mL in sterile PBS. BEVs were further purified by size-exclusion chromatography using qEVoriginal 35 nm columns (Izon Science) according to the manufacturer’s instructions, and fractions containing BEVs were pooled. The pooled fractions were passed through a final 0.22 μm syringe filter (Sartorius). BEV preparations were stored at 4°C, and sterility was confirmed by plating on BHI–hemin agar. Particle size and concentration were determined by nanoparticle tracking analysis using the ZetaView platform (ZetaView, Particle Matrix).

### Nanoparticle tracking analysis (ZetaView)

The size distribution and concentration of bacterial extracellular vesicles were determined by nanoparticle tracking analysis using a ZetaView PMX 220 TWIN instrument (Particle Metrix, Germany) operated according to the manufacturer’s instructions. Vesicle suspensions were diluted up to 50,000-fold in particle-free water to obtain approximately 50–200 particles per frame. For each sample, videos were acquired at 11 positions with two measurement cycles per position (60 frames per position; 2 s video length; temperature 25°C; camera sensitivity 80; shutter 100). Data were analyzed using ZetaView NTA software version 8.05.12, with post-acquisition settings of minimum brightness 20, minimum area 5, maximum area 2,000, and trace length 30.

### Cell lines and culture conditions

B16F10 melanoma and CMT19T lung carcinoma cells were maintained in high-glucose DMEM supplemented with 10% fetal bovine serum and 100 U/mL penicillin/streptomycin. Cells were cultured at 37°C in 5% CO_2_ in flasks coated with 0.1% porcine gelatin.

The human myeloid leukemia cell line THP-1-Blue (Invivogen) expressing an NF-κB-inducible secreted alkaline phosphatase (SEAP) reporter was maintained in RPMI-1640 supplemented with 10% fetal bovine serum, 1% penicillin/streptomycin, 100 μg/mL Normocin, and 10 μg/mL blasticidin. The human embryonic kidney cell line 293 (HEK293) expressing human TLR2 and hTLR4 inducible SEAP reporter (HEK-Blue) was maintained in RPMI-1640 supplemented with 10% fetal bovine serum, 1% penicillin/streptomycin, 100 μg/mL Normocin, and 1 × HEK-Blue selection cocktail.

### THP1-Blue NF-κB reporter assay

THP1-Blue cells were seeded at 1 × 10^5^ cells per well in 96-well plates and incubated for 24 h with BEVs or LPS as a positive control. NF-κB activity was quantified using QUANTI-Blue detection medium according to the manufacturer’s instructions, and absorbance was measured at 650 nm.

### HEK-Blue TLR2 and TLR4 reporter assays

HEK-Blue hTLR2 and HEK-Blue hTLR4 cells were seeded at 5 × 10^4^ cells per well in 96-well plates in HEK-Blue detection medium containing BEVs or control ligands. LTA-BS (from *B. subtilis*; Invivogen) and LPS (from *E. coli* O111:B4; Sigma) were used as positive controls for TLR2 and TLR4, respectively. Cells were incubated for 16 h at 37°C in 5% CO_2_, and reporter activity was quantified by absorbance at 650 nm measured on a VERSAmax microplate reader (Molecular Devices, San Jose, USA).

### Tumor-cell viability assay

To assess whether Bt BEVs directly altered tumor-cell viability, B16F10 cells were seeded at 2,000 cells per well in 96-well plates and allowed to adhere for 24 h. Cells were then treated with a range of BEVs for a further 24 h. alamarBlue reagent was added for 4 h at 37°C in 5% CO_2_, and fluorescence intensity was measured at 590 nm on a VERSAmax microplate reader (Molecular Devices, San Jose, USA).

### Subcutaneous tumor models and BEV treatment

A total of 4 × 10^5^ B16F10 melanoma cells or 1 × 10^6^ CMT19T lung carcinoma cells were injected into the flank of 8–12-week-old male C57BL/6 mice in 100 μL PBS. Tumor volume was measured three times weekly by digital caliper from the onset of palpable growth and calculated as length × width^2^ ×0.52. B16F10 tumors were typically harvested on day 14 and CMT19T tumors on day 18.

For BEV treatment protocols, BEVs were administered either IP three times weekly or IV every 3 days from the onset of palpable tumor, as specified in the corresponding experimental design. For B16F10 route-comparison experiments, 1 × 10^10^ BEVs were delivered by either IP or IV injection. In IV dose-response studies, tumor-bearing mice received escalating BEV doses, with 1 × 10^10^ particles representing the highest and most effective dose tested. The same IV treatment schedule was used in the CMT19T model. Body weight was monitored throughout treatment.

### Experimental B16F10 metastasis model

A total of 1 × 10^6^ B16F10 cells were injected IV via the tail vein. Tumor cells were allowed to circulate and seed at metastatic sites before initiation of BEV therapy. BEV treatment began 3 days after tumor-cell injection, and lungs were harvested on day 14 for quantification of visible pulmonary surface nodule number and average histological pulmonary lesion area. Visible pulmonary surface nodules were counted without blinding to treatment group. Histological pulmonary lesion area was quantified in ImageJ by an investigator blinded to treatment group.

### Tissue histology

To assess treatment-associated tissue toxicity, major organs were collected at endpoint and processed as formaldehyde-fixed paraffin-embedded tissue. Organs were fixed for 16 h in 4% paraformaldehyde at 4°C, dehydrated through graded ethanol, cleared in xylene, and embedded in paraffin. Paraffin blocks were sectioned at 6 μm using a rotary microtome, mounted onto positively charged glass slides, and incubated for 16 h at 37°C. Sections were then deparaffinized in xylene, rehydrated through decreasing concentrations of ethanol into water, and stained with hematoxylin and eosin using a Leica ST5020 tissue multi-stainer. Coverslips were mounted with Neo-Mount. Brightfield images were acquired using an Olympus BX60 microscope fitted with a Jenoptik C10 camera.

### BEV biodistribution

For *in vivo* tracking studies, NanoLuc-tagged Bt BEVs (BEV-NLuc) were generated by genetically inserting nanoluciferase into parental *Bacteroides thetaiotaomicron* VPI-5482, as previously described (11, 23), followed by BEV isolation as described above. For biodistribution experiments, 1 × 10^10^ BEV-NLuc or vehicle control was injected IV via the tail vein into day 10 B16F10 tumor-bearing mice. Mice were sacrificed 3 h post-dose, and organs were excised, immersed in sterile PBS, blotted dry, and arranged in large Petri dishes. Organs were then immersed in 10 mL furimazine substrate (Nano-Glo Luciferase Assay System, Promega) diluted 1:20 in PBS for 5 min before imaging on a Bruker In-Vivo Xtreme instrument using a 1-min bioluminescence exposure setting. Regions of interest were drawn around each organ, and bioluminescent signal was quantified in ImageJ.

### Statistical analysis

Data are presented as mean ± SEM unless otherwise indicated. Statistical analyses were performed using GraphPad Prism 9. Comparisons between two groups were performed using two-tailed unpaired *t*-tests, with Welch’s correction applied where appropriate. Comparisons involving more than two groups were performed using ordinary one-way ANOVA with Dunnett’s multiple-comparisons test against the relevant vehicle control unless otherwise stated. *P* < 0.05 was considered statistically significant. Significance thresholds are indicated as *P < 0.05, ***P < 0.001, and ****P < 0.0001.

## RESULTS

### Intravenous administration is required for robust anti-tumor activity of Bt BEVs

**Fig 1:**
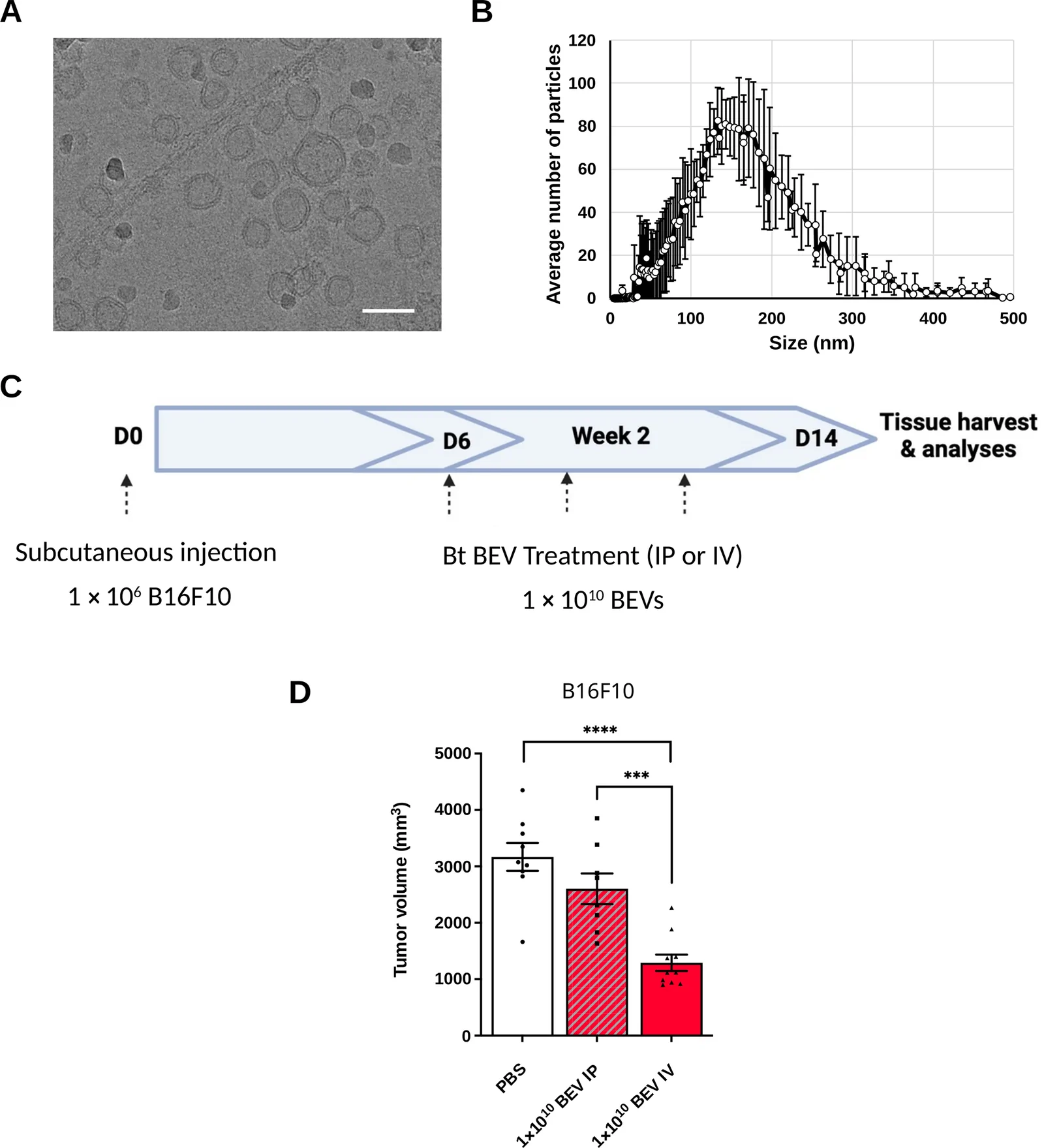
Bt BEVs are nanoscale membrane-bound vesicles and show route-dependent anti-tumor activity. (**A**) Representative cryo-transmission electron micrograph of Bt-derived BEVs generated using the established preparation workflow. Vesicles display rounded, membrane-bound morphology. Image adapted from Juodeikis et al. (19) (CC BY 4.0). Scale bar, 100 nm. (**B**) Nanoparticle tracking analysis (NTA) size distribution of Bt-derived BEVs. Particle size (nm) is plotted against the average number of particles per size bin (± SD) for three independent samples prepared on different days. Across the three biological preparations, the mean number-weighted median (D50) diameter was 142 ± 27 nm, and the mean particle diameter was 153 ± 29 nm (mean ± SD). (**C**) Experimental outline of the B16F10 primary melanoma route-comparison experiment. Mice were inoculated subcutaneously with 1 × 10^6^ B16F10 cells on day 0 and treated with 1 × 10^10^ Bt BEVs by either intraperitoneal (IP) or intravenous (IV) administration on experimental days 6, 9, and 11, followed by tissue harvest and analysis on day 14. (**D**) Quantification of endpoint B16F10 tumor volume following vehicle control, IP BEV, or IV BEV treatment. Bars represent mean ± SEM, *n* = 8–10. Statistical significance was calculated by a two-tailed unpaired *t*-test. IP vs PBS, *P* = 0.1447; IV vs PBS, *P* < 0.0001; IV vs IP, *P* = 0.0004. ***P < 0.001, ****P < 0.0001.

To define the vesicle preparation used for downstream functional testing, we first assessed the morphology and size distribution of Bt BEVs. Consistent with previous cryo-TEM characterization of Bt BEVs generated using the same established preparation workflow, representative vesicles displayed rounded, membrane-bound morphology (Fig. 1A). Across three independent Bt BEV preparations generated on different days, nanoparticle tracking analysis showed a mean number-weighted median (D50) diameter of 142 ± 27 nm and a mean particle diameter of 153 ± 29 nm (mean ± SD across biological preparations; Fig. 1B). Together, these data support the recovery of nanoscale Bt BEV preparations suitable for *in vivo* functional testing.

To evaluate the therapeutic potential of Bt BEVs *in vivo*, we next assessed whether the delivery route influences anti-tumor efficacy. Bt BEVs were administered either IP or IV at an equivalent dose (1 × 10¹⁰ BEVs) in mice bearing established B16F10 melanoma tumors (Fig. 1C). While IP administration produced a non-significant trend reduction in tumor burden (*P* = 0.144), IV delivery resulted in a marked suppression of tumor growth relative to both PBS control (*P* < 0.0001) and IP-treated animals (*P* = 0.0004) (Fig. 1D). Quantification of endpoint tumor volumes demonstrated a substantially greater reduction following IV administration relative to IP treatment, indicating that delivery route is a key determinant of BEV efficacy. Under the tested conditions, IV administration produced greater anti-tumor activity than IP administration.

### Intravenously delivered Bt BEVs suppress primary tumor growth in a dose-dependent manner

**Fig 2:**
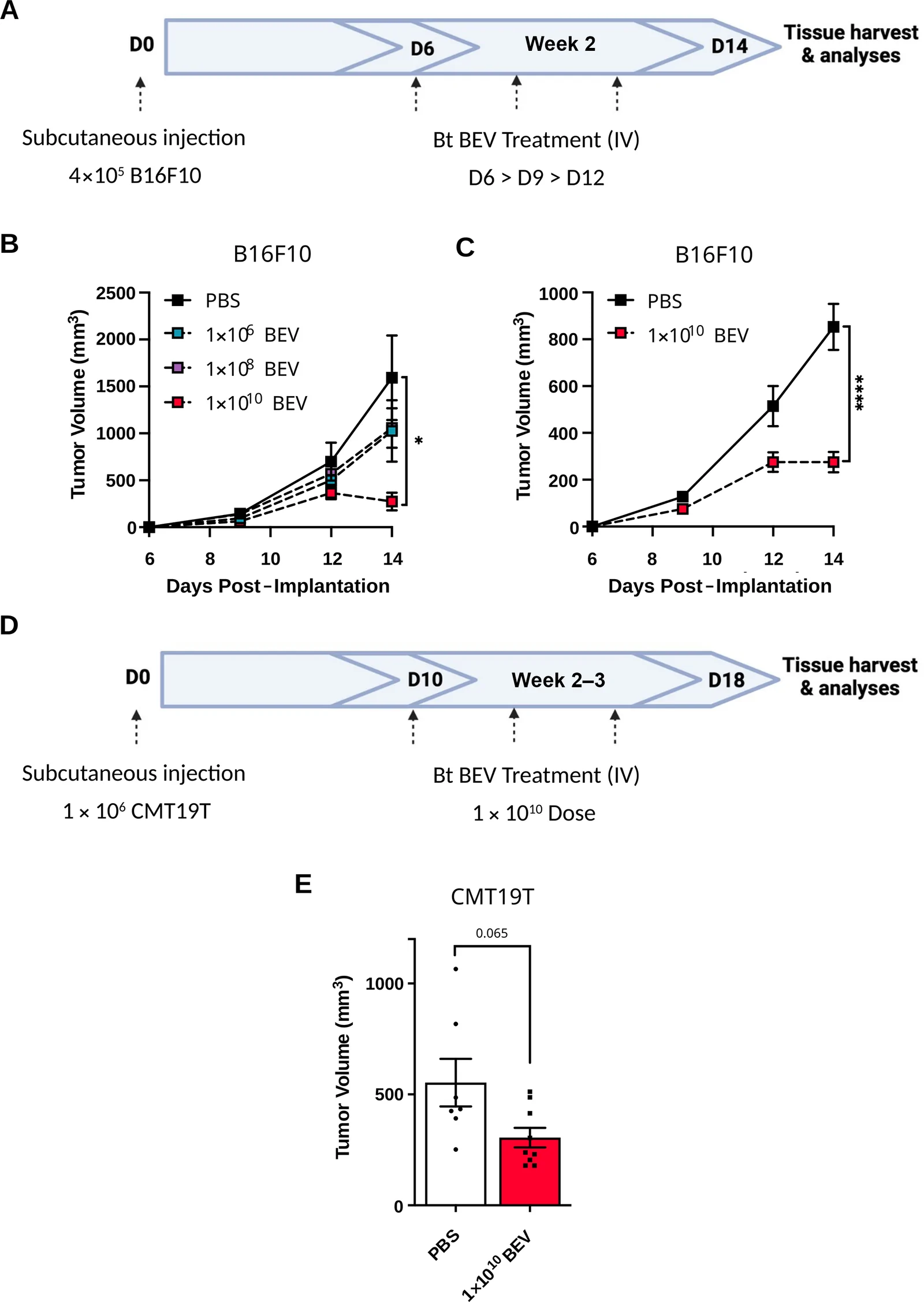
IV Bt BEVs suppress primary tumor growth in a dose-dependent manner. (**A**) Experimental outline of the B16F10 primary melanoma dose-response study. Mice were inoculated subcutaneously with 4 × 105 B16F10 cells on day 0 and treated IV with Bt BEVs at 3-day intervals from the onset of palpable tumor (days 6, 9, and 12), followed by tissue harvest and analysis on day 14. (**B**) Mean ± SEM B16F10 tumor growth over time following IV administration of the indicated BEV doses (*n* = 5). Endpoint dose-response comparisons were analyzed by ordinary one-way ANOVA with Dunnett’s multiple-comparisons test against the PBS control. Adjusted *P* values: 1 × 10⁶ BEVs, *P* = 0.4286; 1 × 10⁸ BEVs, *P* = 0.4706; 1 × 10¹⁰ BEVs, *P* = 0.0185. (**C**) Mean ± SEM B16F10 tumor growth over time following IV administration of a defined 1 × 10^10^ BEV dose (*n* = 21–23, pooled across three independent experiments). Endpoint tumor volume was analyzed by Welch’s *t*-test, *P* < 0.0001. (**D**) Experimental outline of the CMT19T primary tumor study. Mice were inoculated subcutaneously with 1 × 106 CMT19T cells on day 0 and treated IV with 1 × 10^10^ Bt BEVs from the onset of palpable tumor, followed by tissue harvest and analysis on day 18. (**E**) Mean ± SEM endpoint CMT19T tumor volume following IV administration of 1 × 10^10^ Bt BEVs (*n* = 7–9). Endpoint tumor volume was analyzed by Welch’s *t*-test, *P* = 0.0653. *P < 0.05, ****P < 0.0001.

We next evaluated the therapeutic efficacy of Bt BEVs in primary tumor models. Mice bearing established B16F10 melanoma tumors were treated with increasing doses of BEVs administered IV at 3-day intervals following tumor palpation (Fig. 2A). IV administration of Bt BEVs resulted in dose-dependent inhibition of tumor growth, with the highest dose tested (1 × 10¹⁰ BEVs) producing the most pronounced effect (*P* = 0.0185) (Fig. 2B). Tumor growth curves showed a reduction in tumor progression across the treatment period, and endpoint tumor volumes across multiple independent experiments were significantly decreased in the high-dose group relative to controls (*P* < 0.0001) (Fig. 2C).

We also tested the same dosing regimen in the CMT19T lung carcinoma model. BEV-treated animals showed a non-significant trend toward reduced endpoint tumor volume compared with controls (*P* = 0.0653; Fig. 2D and E). Bt BEV treatment was not associated with overt body weight loss, and representative H&E-stained sections of major organs showed preserved tissue architecture without obvious inflammatory or necrotic changes (Fig. S1). Together, these data show reduced primary B16F10 melanoma growth after IV Bt BEV treatment, with a smaller, non-significant trend toward reduced tumor burden in the CMT19T model. No obvious histological evidence of treatment-associated tissue damage was observed in the representative organ sections examined.

### Bt BEVs inhibit metastatic tumor outgrowth in a preclinical melanoma model

**Fig 3:**
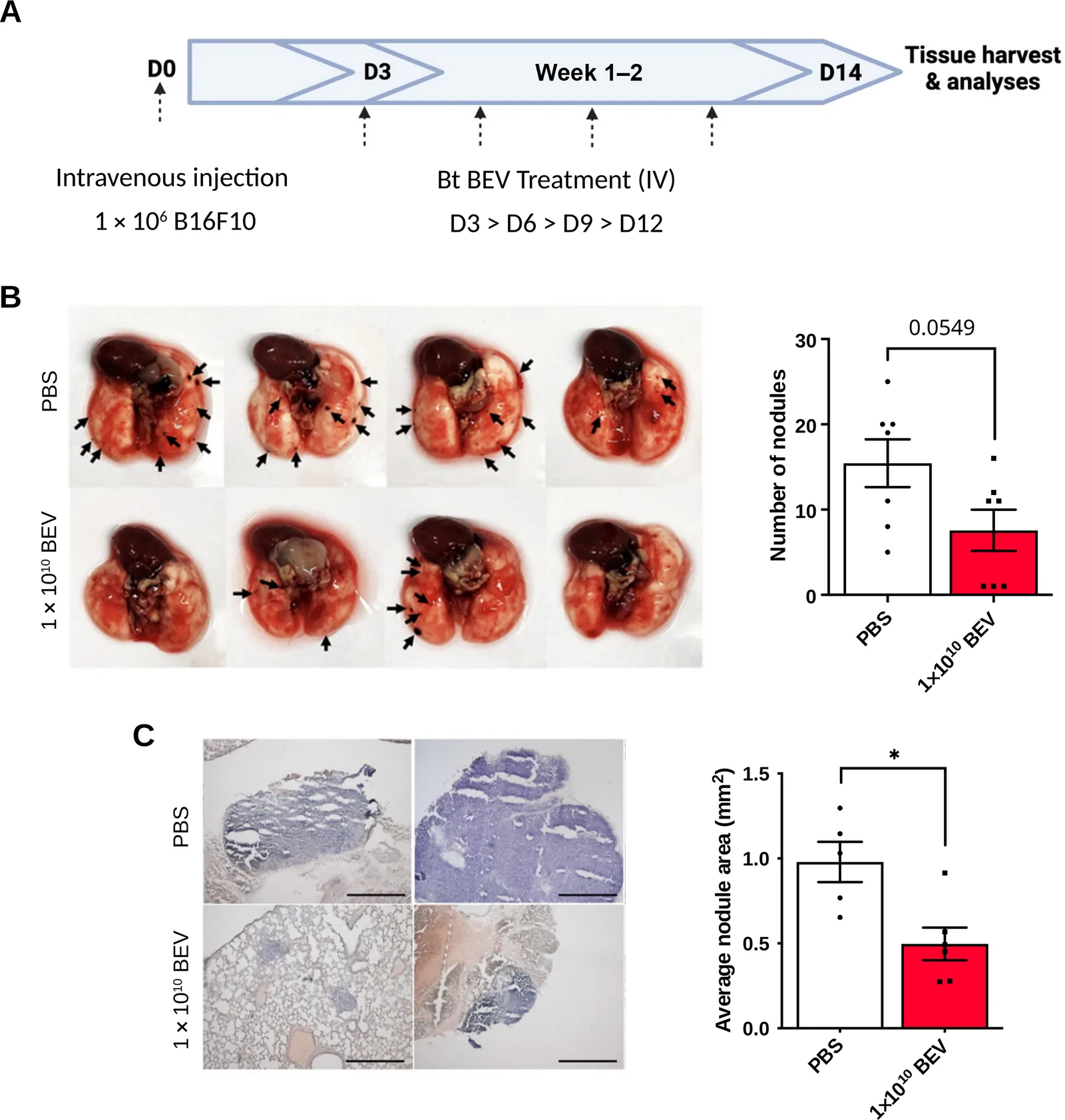
Bt BEVs reduce pulmonary lesion burden in a melanoma experimental metastasis model. (**A**) Experimental outline of the B16F10 experimental metastasis study. Mice were injected IV with 1 × 10^6^ B16F10 cells on day 0 and allowed to seed in the lungs for 3 days before initiation of IV Bt BEV therapy (1 × 10^10^ BEVs) on days 3, 6, 9, and 12, followed by tissue harvest and analysis on day 14. (**B**) Representative lung images (left) and quantification of the number of visible pulmonary surface lesions (right) following vehicle control or Bt BEV treatment. Bars represent mean ± SEM, *n* = 7. Two-tailed unpaired *t*-test, *P* = 0.0549. (**C**) Representative H&E-stained lung sections acquired at 4× objective (left) and quantification of average pulmonary lesion area (right) in animals treated with vehicle control or 1 × 10^10^ Bt BEVs. Scale bars: 200 µm. Lesion area was quantified in ImageJ by an investigator blinded to treatment group, with each mouse contributing one mean lesion-area value to the analysis. Bars represent mean ± SEM, *n* = 5–6. Two-tailed unpaired *t*-test, *P* = 0.0110. *P < 0.05.

Given the clinical importance of metastatic progression in melanoma, we next investigated whether Bt BEV treatment impacts secondary tumor outgrowth. To model metastatic disease, B16F10 melanoma cells were introduced IV to allow seeding in the lung, followed by initiation of BEV treatment after tumor-cell dissemination (Fig. 3A). At the experimental endpoint, visual inspection of lung tissue revealed fewer and smaller pulmonary nodules in BEV-treated animals compared to controls. Quantitative analysis showed a numerical reduction in visible pulmonary surface lesions that approached statistical significance (*P* = 0.0549), while histological analysis demonstrated a significant reduction in mean pulmonary lesion area (*P* = 0.011) (Fig. 3B and C). Together, these findings are consistent with Bt BEV treatment reducing pulmonary tumor outgrowth after initial tumor-cell seeding. These results extend the observed anti-tumor activity of Bt BEVs beyond the primary subcutaneous tumor setting.

### Bt BEVs activate human innate immune reporter systems and are detected in tumor tissue following systemic administration

**Fig 4:**
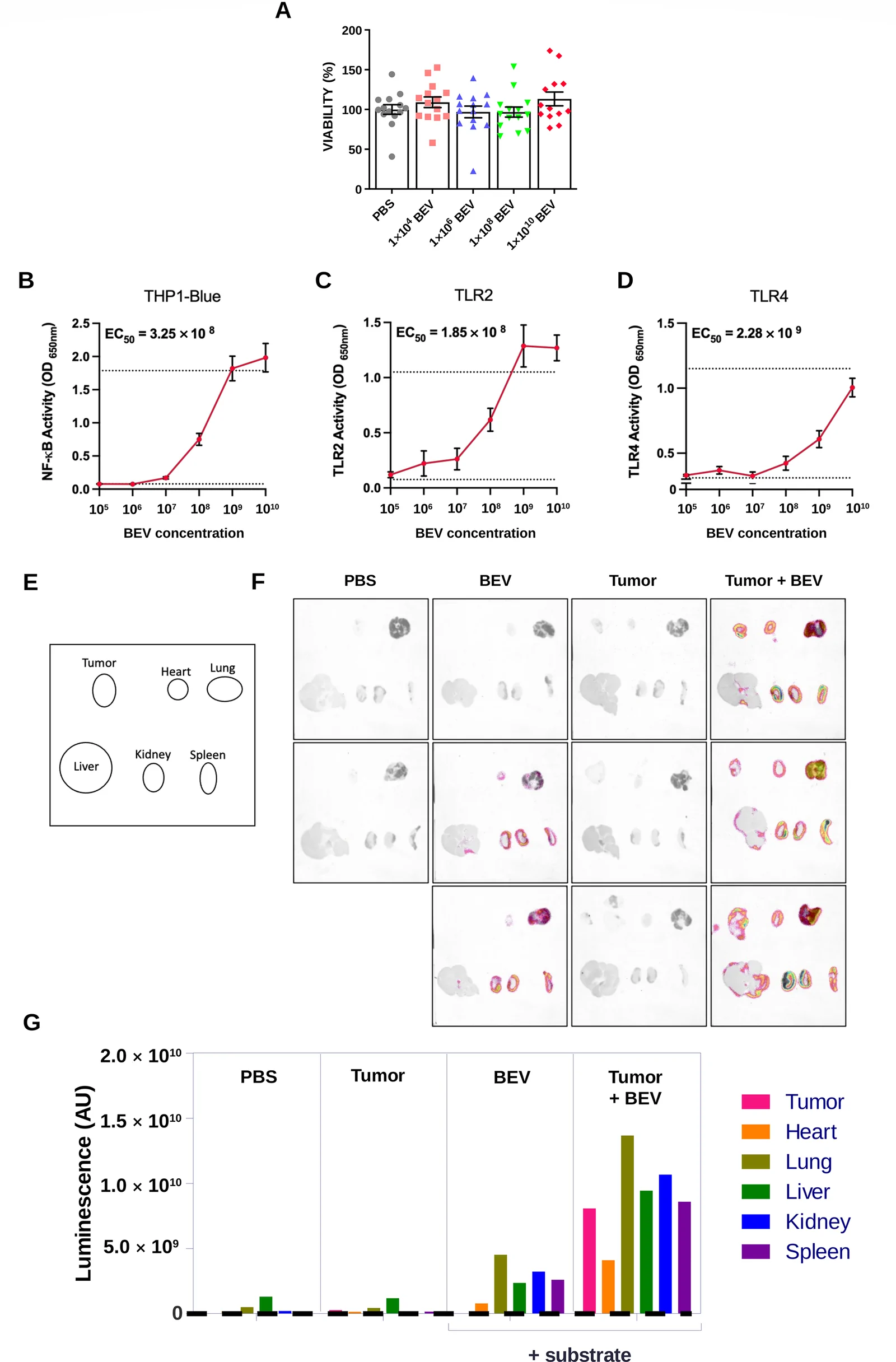
Bt BEVs activate human innate immune reporter systems and are detected in tumor tissue. (**A**) Relative alamarBlue fluorescence from B16F10 cells after 24-h *in vitro* treatment with the indicated BEV doses, with *n* = 14 technical replicates across *N* = 3 independent experiments (five technical replicates per experiment). Statistical significance was calculated by ordinary one-way ANOVA; no significant difference was detected among treatment groups, *P* = 0.3425. (**B**) Quantification of NF-κB activity in THP1-Blue monocytes in response to escalating BEV doses. Activity was measured by QUANTI-Blue detection of the SEAP reporter, *n* = 5. (**C**) Quantification of TLR2 activity in HEK-Blue hTLR2 cells in response to escalating BEV doses, *n* = 3. (**D**) Quantification of TLR4 activity in HEK-Blue hTLR4 cells in response to escalating BEV doses, *n* = 3. (**E**) Schematic showing the *ex vivo* organ layout used for biodistribution imaging. (**F**) Representative *ex vivo* bioluminescence images of organs from non-tumor-bearing or day 10 B16F10 tumor-bearing mice following IV administration of vehicle control or 1 × 10^10^ NanoLuc-tagged BEVs (BEV-NLuc). (**G**) Quantification of absolute bioluminescent signal across indicated organs following vehicle or BEV-NLuc administration, *n* = 3. For biodistribution studies, organs were harvested 3 h after IV dosing and imaged following incubation in furimazine substrate.

To explore potential mechanisms underlying the observed anti-tumor effects, we first assessed whether Bt BEVs directly impact tumor-cell viability. Co-culture of B16F10 cells with increasing concentrations of BEVs did not alter alamarBlue fluorescence after 24 h (*P* = 0.342; Fig. 4A), indicating no reduction *in B16F10* metabolic viability under these *in vitro* conditions. We next evaluated the capacity of Bt BEVs to stimulate innate immune signaling pathways. BEV co-culture with human THP-1-Blue cells increased NF-κB reporter activity in a dose-dependent manner (Fig. 4B). Bt BEVs also increased SEAP reporter activity in human HEK-Blue hTLR2 and hTLR4 cells, with a larger response in the hTLR2 reporter line under the tested assay conditions (Fig. 4C and D). Finally, to determine whether systemically administered BEVs can access tumor tissue, we performed biodistribution studies using NanoLuc-expressing Bt BEVs (BEV-NLuc). Following IV administration, NanoLuc-associated signal was detected in multiple excised organs, including tumor tissue (Fig. 4E through G). Together, these findings show that Bt BEVs activate human innate immune reporter systems *in vitro* and that labeled BEV preparations are detected in tumor-containing tissue following systemic administration.

## DISCUSSION

Our data show that BEVs derived from the human intestinal commensal *Bacteroides thetaiotaomicron* exert anti-tumor activity *in vivo* that is strongly influenced by delivery route. In the preclinical melanoma model examined here, IV administration, but not IP delivery, produced marked suppression of primary B16F10 melanoma growth and reduced pulmonary lesion burden in an experimental metastasis model. Bt BEV preparations did not reduce B16F10 metabolic viability during the tested 24-h *in vitro* exposure, indicating that the preparations did not produce measurable loss of metabolic viability under these conditions. Prior anti-cancer BEV studies have utilized non-commensal or engineered systems, making the present study distinct in its focus on native vesicles from a human commensal bacterium (12, 24).

A key conclusion of this study is that the delivery route is a major determinant of Bt BEV anti-tumor efficacy. This finding aligns with the broader BEV literature showing that biological activity depends not only on vesicle dose or source organism, but also on the route by which vesicles encounter host tissues and immune compartments. In cancer models, Kim et al. (12) showed that IV-administered BEVs from attenuated pathogens accumulate in tumors and induce anti-tumor immune responses. By contrast, engineered anti-EGFR BEVs derived from non-pathogenic *E. coli* BL21(DE3) have shown anti-tumor activity after IP administration in a 4T1 breast cancer model (24), while bioengineered commensal-derived Bt BEVs delivered orally or intranasally can elicit local and systemic immune responses in vaccine settings (20, 25). Recent preclinical cancer studies have further shown that orally delivered commensal or engineered BEVs can also elicit anti-tumor responses through distinct mucosal or vaccine-like mechanisms (26, 27). Thus, the optimal route for BEV-based therapeutics is likely context-dependent, reflecting differences in vesicle source, engineering strategy, cargo, tissue exposure, and intended immune mechanism. In our model, IV delivery was superior to IP delivery, but the pharmacokinetic and cellular basis of this route dependence remains to be defined.

Although the present study does not define the host mechanisms responsible for tumor control, the *in vitro* and biodistribution findings identify plausible directions for further investigation. Bt BEVs activated human NF-κB, TLR2, and TLR4 reporter systems *in vitro*, and NanoLuc-associated signal from labeled BEV preparations was detected in excised tumor tissue after IV administration. These findings show that Bt BEV preparations contain ligands capable of engaging innate immune reporter pathways and can reach tumor-containing tissue, but do not establish which receptor or host-cell population is required for the *in vivo* effect. Previous studies showing that Bt BEVs modulate dendritic-cell and THP-1-Blue responses in non-cancer settings provide biological context for these observations, rather than direct evidence for the mechanism operating in the present tumor model (8, 16).

The strongest efficacy data were generated in B16F10 melanoma, while the CMT19T experiment showed a smaller, non-significant reduction in tumor burden. Further studies will therefore be required to determine how broadly Bt BEV activity extends across tumor types and to identify the active BEV components and host pathways responsible for tumor *control*. Nonetheless, this work provides proof-of-principle that IV-administered native commensal Bt BEVs can suppress primary B16F10 melanoma growth and reduce pulmonary tumor burden *in vivo*, while identifying delivery route as a major determinant of activity under the tested conditions. More broadly, these findings support further evaluation of microbiome-derived vesicles as candidate cell-free therapeutic platforms.

## Data Availability

The numerical source data underlying the quantitative analyses presented in this study are provided in the supplemental material. Additional data supporting the findings of this study are available from the corresponding authors upon reasonable request.

## SUPPLEMENTAL MATERIAL

Fig. S1 legend spectrum.02512-26-s0001.docx Bt BEV administration is not associated with significant weight loss or histopathological abnormalities in major organs. (A) Mean ± SEM body weight change over time in B16F10 tumor-bearing mice treated with the indicated BEV doses, *n* = 5. (B) Representative H&E-stained sections of lungs, liver, and spleens from vehicle control or 1 × 10^10^ Bt BEV-treated animals, acquired using a 4× objective. Scale bars: 200 µm.

File S1 spectrum.02512-26-s0002.xlsx Raw data.

Fig. S1 spectrum.02512-26-s0003.tif Bt BEV administration is not associated with significant weight loss or histopathological abnormalities in major organs.

ASM does not own the copyrights to Supplemental Material that may be linked to, or accessed through, an article. The authors have granted ASM a non-exclusive, world-wide license to publish the Supplemental Material files. Please contact the corresponding author directly for reuse.

## References

[1] Routy B, Le Chatelier E, Derosa L, Duong CPM, Alou MT, Daillère R, Fluckiger A, Messaoudene M, Rauber C, Roberti MP, et al.. 2018. Gut microbiome influences efficacy of PD-1-based immunotherapy against epithelial tumors. Science 359:91–97. doi:10.1126/science.aan3706 29097494

[2] Gopalakrishnan V, Spencer CN, Nezi L, Reuben A, Andrews MC, Karpinets TV, Prieto PA, Vicente D, Hoffman K, Wei SC, et al.. 2018. Gut microbiome modulates response to anti-PD-1 immunotherapy in melanoma patients. Science 359:97–103. doi:10.1126/science.aan4236 PMC5827966 29097493

[3] Matson V, Fessler J, Bao R, Chongsuwat T, Zha Y, Alegre M-L, Luke JJ, Gajewski TF. 2018. The commensal microbiome is associated with anti-PD-1 efficacy in metastatic melanoma patients. Science 359:104–108. doi:10.1126/science.aao3290 PMC6707353 29302014

[4] Blake SJ, Wolf Y, Boursi B, Lynn DJ. 2024. Role of the microbiota in response to and recovery from cancer therapy. Nat Rev Immunol 24:308–325. doi:10.1038/s41577-023-00951-0 37932511

[5] Elkrief A, Pidgeon R, Maleki Vareki S, Messaoudene M, Castagner B, Routy B. 2025. The gut microbiome as a target in cancer immunotherapy: opportunities and challenges for drug development. Nat Rev Drug Discov 24:685–704. doi:10.1038/s41573-025-01211-7 40457025

[6] Gujrati V, Kim S, Kim S-H, Min JJ, Choy HE, Kim SC, Jon S. 2014. Bioengineered bacterial outer membrane vesicles as cell-specific drug-delivery vehicles for cancer therapy. ACS Nano 8:1525–1537. doi:10.1021/nn405724x 24410085

[7] Liu G, Ma N, Cheng K, Feng Q, Ma X, Yue Y, Li Y, Zhang T, Gao X, Liang J, Zhang L, Wang X, Ren Z, Fu Y-X, Zhao X, Nie G. 2024. Bacteria-derived nanovesicles enhance tumour vaccination by trained immunity. Nat Nanotechnol 19:387–398. doi:10.1038/s41565-023-01553-6 38052943

[8] Durant L, Stentz R, Noble A, Brooks J, Gicheva N, Reddi D, O’Connor MJ, Hoyles L, McCartney AL, Man R, Pring ET, Dilke S, Hendy P, Segal JP, Lim DNF, Misra R, Hart AL, Arebi N, Carding SR, Knight SC. 2020. Bacteroides thetaiotaomicron-derived outer membrane vesicles promote regulatory dendritic cell responses in health but not in inflammatory bowel disease. Microbiome 8:88. doi:10.1186/s40168-020-00868-z PMC7282036 32513301

[9] Chelakkot C, Choi Y, Kim D-K, Park HT, Ghim J, Kwon Y, Jeon J, Kim M-S, Jee Y-K, Gho YS, Park H-S, Kim Y-K, Ryu SH. 2018. *Akkermansia muciniphila*-derived extracellular vesicles influence gut permeability through the regulation of tight junctions. Exp Mol Med 50:e450. doi:10.1038/emm.2017.282 PMC5903829 29472701

[10] Gilmore WJ, Johnston EL, Bitto NJ, Zavan L, O’Brien-Simpson N, Hill AF, Kaparakis-Liaskos M. 2022. *Bacteroides fragilis* outer membrane vesicles preferentially activate innate immune receptors compared to their parent bacteria. Front Immunol 13:970725. doi:10.3389/fimmu.2022.970725 PMC9592552 36304461

[11] Jones EJ, Parker A, Juodeikis R, Blackshaw LA, Brion A, Carding SR. 2025. Special delivery - extracellular vesicles released by commensal gut bacteria deliver bioactive protein to distal organs. Extracell Vesicles Circ Nucl Acids 6:791–806. doi:10.20517/evcna.2025.32 PMC12809395 41551599

[12] Kim OY, Park HT, Dinh NTH, Choi SJ, Lee J, Kim JH, Lee S-W, Gho YS. 2017. Bacterial outer membrane vesicles suppress tumor by interferon-γ-mediated antitumor response. Nat Commun 8:626. doi:10.1038/s41467-017-00729-8 PMC5606984 28931823

[13] Hooper LV, Wong MH, Thelin A, Hansson L, Falk PG, Gordon JI. 2001. Molecular analysis of commensal host-microbial relationships in the intestine. Science 291:881–884. doi:10.1126/science.291.5505.881 11157169

[14] Xu J, Bjursell MK, Himrod J, Deng S, Carmichael LK, Chiang HC, Hooper LV, Gordon JI. 2003. A genomic view of the human-*Bacteroides thetaiotaomicron* symbiosis. Science 299:2074–2076. doi:10.1126/science.1080029 12663928

[15] Sonnenburg JL, Xu J, Leip DD, Chen C-H, Westover BP, Weatherford J, Buhler JD, Gordon JI. 2005. Glycan foraging *in vivo* by an intestine-adapted bacterial symbiont. Science 307:1955–1959. doi:10.1126/science.1109051 15790854

[16] Fonseca S, Carvalho AL, Miquel-Clopés A, Jones EJ, Juodeikis R, Stentz R, Carding SR. 2022. Extracellular vesicles produced by the human gut commensal bacterium *Bacteroides thetaiotaomicron* elicit anti-inflammatory responses from innate immune cells. Front Microbiol 13:1050271. doi:10.3389/fmicb.2022.1050271 PMC9684339 36439842

[17] Jones EJ, Booth C, Fonseca S, Parker A, Cross K, Miquel-Clopés A, Hautefort I, Mayer U, Wileman T, Stentz R, Carding SR. 2020. The uptake, trafficking, and biodistribution of *Bacteroides thetaiotaomicron* generated outer membrane vesicles. Front Microbiol 11:57. doi:10.3389/fmicb.2020.00057 PMC7015872 32117106

[18] Modasia AA, Jones EJ, Martel L-P, Louvel H, Couraud P-O, Blackshaw LA, Carding SR. 2023. The use of a multicellular *in vitro* model to investigate uptake and migration of bacterial extracellular vesicles derived from the human gut commensal *Bacteroides thetaiotaomicron*. J Extracell Biol 2:e93. doi:10.1002/jex2.93 PMC11080816 38939073

[19] Juodeikis R, Martins C, Saalbach G, Richardson J, Koev T, Baker DJ, Defernez M, Warren M, Carding SR. 2024. Differential temporal release and lipoprotein loading in *B. thetaiotaomicron* bacterial extracellular vesicles. J Extracell Vesicles 13:e12406. doi:10.1002/jev2.12406 PMC10797578 38240185

[20] Carvalho AL, Fonseca S, Miquel-Clopés A, Cross K, Kok K-S, Wegmann U, Gil-Cordoso K, Bentley EG, Al Katy SHM, Coombes JL, Kipar A, Stentz R, Stewart JP, Carding SR. 2019. Bioengineering commensal bacteria-derived outer membrane vesicles for delivery of biologics to the gastrointestinal and respiratory tract. J Extracell Vesicles 8:1632100. doi:10.1080/20013078.2019.1632100 PMC6598475 31275534

[21] Juodeikis R, Jones E, Deery E, Beal DM, Stentz R, Kräutler B, Carding SR, Warren MJ. 2022. Nutrient smuggling: Commensal gut bacteria-derived extracellular vesicles scavenge vitamin B12 and related cobamides for microbe and host acquisition. J Extracell Biol 1:e61. doi:10.1002/jex2.61 PMC11080852 38939214

[22] Stentz R, Miquel-Clopés A, Carding SR. 2022. Production, isolation, and characterization of bioengineered bacterial extracellular membrane vesicles derived from bacteroides thetaiotaomicron and their use in vaccine development. Methods Mol Biol 2414:171–190. doi:10.1007/978-1-0716-1900-1_11 34784038

[23] Huang Y, Beringhs AO, Chen Q, Song D, Chen W, Lu X, Fan T-H, Nieh M-P, Lei Y. 2019. Genetically engineered bacterial outer membrane vesicles with expressed nanoluciferase reporter for *in vivo* bioluminescence kinetic modeling through noninvasive imaging. ACS Appl Bio Mater 2:5608–5615. doi:10.1021/acsabm.9b00690 35021555

[24] Rezaei Adriani R, Mousavi Gargari SL, Bakherad H, Amani J. 2023. Anti-EGFR bioengineered bacterial outer membrane vesicles as targeted immunotherapy candidate in triple-negative breast tumor murine model. Sci Rep 13:16403. doi:10.1038/s41598-023-43762-y PMC10541432 37775519

[25] Carvalho AL, Miquel-Clopés A, Wegmann U, Jones E, Stentz R, Telatin A, Walker NJ, Butcher WA, Brown PJ, Holmes S, Dennis MJ, Williamson ED, Funnell SGP, Stock M, Carding SR. 2019. Use of bioengineered human commensal gut bacteria-derived microvesicles for mucosal plague vaccine delivery and immunization. Clin Exp Immunol 196:287–304. doi:10.1111/cei.13301 PMC6514708 30985006

[26] Yue Y, Xu J, Li Y, Cheng K, Feng Q, Ma X, Ma N, Zhang T, Wang X, Zhao X, Nie G. 2022. Antigen-bearing outer membrane vesicles as tumour vaccines produced in situ by ingested genetically engineered bacteria. Nat Biomed Eng 6:898–909. doi:10.1038/s41551-022-00886-2 35501399

[27] Preet R, Islam MA, Shim J, Rajendran G, Mitra A, Vishwakarma V, Kutz C, Choudhury S, Pathak H, Dai Q, Sun W, Madan R, Zhong C, Markiewicz MA, Zhang J. 2025. Gut commensal bifidobacterium-derived extracellular vesicles modulate the therapeutic effects of anti-PD-1 in lung cancer. Nat Commun 16:3500. doi:10.1038/s41467-025-58553-4 PMC11993705 40221398

