# Supplementary material for: Systemically delivered Bacteroides thetaiotaomicron-derived bacterial extracellular vesicles inhibit primary and metastatic melanoma growth
Source: Microbiol Spectr. 2026 Sep 29;14(11):e02512-26. doi: 10.1128/spectrum.02512-26 (PMC13729168; doi:10.1128/spectrum.02512-26)
Supplement: Supplementary file 1 [file spectrum.02512-26-s0001.docx]

**Supplementary figure legends**

**Supplementary Figure 1. Bt BEV administration is not associated with significant weight loss or histopathological abnormalities in major organs.**
**(A)** Mean ± SEM body weight change over time in B16F10 tumour-bearing mice treated with the indicated BEV doses, **n = 5**. **(B)** Representative H&E-stained sections of lungs, liver, and spleens from vehicle control or **1 × 10^10^** Bt BEV-treated animals, acquired using a 4× objective. Scale bars, 200 µm.
